# On evaluating health centers groups in Lisbon and Tagus Valley: efficiency, equity and quality

**DOI:** 10.1186/1472-6963-13-529

**Published:** 2013-12-21

**Authors:** Cláudia Ferreira, Rui C Marques, Paulo Nicola

**Affiliations:** 1 Instituto Superior Técnico, University of Lisbon, Lisbon, Portugal; 2 Faculty of Medicine, University of Lisbon, Lisbon, Portugal

**Keywords:** Health center groups, Data envelopment analysis, Efficiency, Quality, Equity, Portugal

## Abstract

**Background:**

Bearing in mind the increasing health expenses and their weight in the Portuguese gross domestic product, it is of the utmost importance to evaluate the performance of Primary Health Care providers taking into account both efficiency, quality and equity. This paper aims to contribute to a better understanding of the performance of Primary Health Care by measuring it in a Portuguese region (Lisbon and Tagus Valley) and identifying best practices. It also intends to evaluate the quality and equity provided.

**Methods:**

For the purpose of measuring the efficiency of the health care centers (ACES) the non-parametric full frontier technique of data envelopment analysis (DEA) was adopted. The recent partial frontier method of order-m was also used to estimate the influence of exogenous variables on the efficiency of the ACES. The horizontal equity was investigated by applying the non-parametric Kruskal-Wallis test with multiple comparisons. Moreover, the quality of service was analyzed by using the ratio between the complaints and the total activity of the ACES.

**Results:**

On the whole, a significant level of inefficiency was observed, although there was a general improvement in efficiency between 2009 and 2010. It was found that nursing was the service with the lowest scores. Concerning the horizontal equity, the analysis showed that there is no evidence of relevant disparities between the different subregions(NUTS III). Concerning the exogenous variables, the purchasing power, the percentage of patients aged 65 years old or older and the population size affect the efficiency negatively.

**Conclusions:**

This research shows that better usage of the available resources and the creation of a learning network and dissemination of best practices will contribute to improvements in the efficiency of the ACES while maintaining or even improving quality and equity. It was also proved that the market structure does matter when efficiency measurement is addressed.

## Background

### Introduction

Healthcare is one of the most important areas for citizens. It is also an area where countries spend a significant part of their resources. For this reason, measuring efficiency becomes relevant. In fact, in 2009 it was estimated that the waste of financial resources with the Portuguese Health System was about 25% of the amount allocated to health [1]. Furthermore, in line with other developed countries, an increase on health expenses, both public and private, has been observed. The growth rate of these expenses even exceeded the growth rate of the GDP and between 2000 and 2008 it rose almost 50%. This growth largely rests on the changes of the Portuguese demographic structure [2]. This situation has created a constant concern about the sustainability of the Portuguese health system [2] so that it becomes necessary to promote efficiency and innovation by adapting management practices and using financial resources in an optimized way.

Despite the fact that the Portuguese health care model is still very dependent on the secondary and differentiated care, the idea that health care systems based on a solid structure of Primary Health Care (PHC) are more cost-effective is, nowadays, fairly pacific [3]. Due to this fact, the Portuguese Primary Health Care has suffered many developments. Additionally, given the current economic situation of Portugal, there is an extra need to evaluate the performance of the public sector and provide value for public money. Health Centers Groups (ACES) productive behavior, and particularly, their efficiency might contribute to this purpose. As stated by Jacobs et al. [4], the efficiency study of health institutions must be a central objective to reduce the public expenditure. According to these authors, the non-parametric frontier technique of Data Envelopment Analysis (DEA) might be an extremely useful tool for estimating the PHC efficiency.

Since the mid-1980s, efficiency in health care has been studied by many authors, both at national and international levels. The first application of DEA in the health sector dates back to 1983, in which Nunamaker and Lewin [5] measured the efficiency of the routine nursing service. Since then, DEA has been widely used in the assessment of health care services efficiency. However, the efficiency of PHC has not been a significant priority. Actually, in the literature review performed in this research, only 9 studies were found until 2002 regarding PHC efficiency [6].

The first study on the efficiency of PHC was published in 1989, when Sexton et al. [7] evaluated the managerial efficiency of veterans administration medical centers. The research concluded that around one third of the total number of health centers were inefficient and that the elimination of the referred inefficiency could reduce annual costs by 300 millions of dollars.

After that, some studies were performed, including: Huang and McLaughlin [8], Pinillos and Antoñanzas [9], Linna et al. [10], Akazili et al. [11], Kirigia et al. [12], Amado and Santos [13], Sebastian and Lemma [14], Halsteinli et al. [15] and Nuti et al. [16].

A common feature of most of these studies, given the level of health care studied, is the variables chosen. The most frequent inputs are the staff and the expenditure, while the most adopted outputs are the different kinds of consultations related to PHC. Nevertheless, while the oldest studies only focused on efficiency, the most recent ones also take into account the factors that affect efficiency.

Concerning quality, the relationship between efficiency and quality of care has had mixed results in prior studies. For example, Helling et al. [17] discovered that increasing efficiency would result in higher quality. On the other hand, Singaroyan et al. [18] found that improving quality of health care may not always lead to efficient operations.

Regarding horizontal equity, it is concerned with fairness, which means equal treatment of patients [19]. In the healthcare field, it measures whether patients from different groups have similar access to the services they equally need.

In Portugal, there are few studies about the performance of PHC, either related to efficiency, quality or equity. Most of the time, evaluation in Portuguese public services involves rankings, classifications and targets. In fact, Amado and Santos’ study [13] is one of the very few that uses DEA in this scope. So, this study contributes to the literature on several grounds. First, it provides an analysis of efficiency, equity and quality of the PHC in Portugal, which is still an area where big improvements must be made. Second, because at the date of the study of Amado and Santos [13], the PHC in Portugal was not organized as it is now, thus the study of efficiency, equity and quality of the ACES of Lisbon and Tagus Valley (LVT) is of the utmost importance when taking into account the current market structure.

### Primary health care in Portugal

#### ***Overview***

Currently, the Portuguese healthcare system is characterized by three coexisting, overlapping systems: the National Health Service (NHS); public and private insurance schemes for certain professions and private voluntary health insurance. The majority of the population receives health care from the NHS, which was founded in 1979 and aims to provide health care, almost free at the point of delivery, with universal coverage being funded mainly by general taxation. Although centrally financed by the Ministry of Health, the NHS comprises five health administrations: North, Centre, Lisbon and Tagus Valley, Alentejo and Algarve. Their main responsibilities are the development of strategic guidelines; coordination of all aspects of health care provision; supervision of management of hospitals and PHC; establishment of agreements and protocols with private bodies and development of a long-term care network [20].

Now, it is important to distinguish between Primary Care and PHC. Primary Care provides entry into the system for all new needs and problems along with people-focused (not disease-oriented) care over time and care for all but with very uncommon or unusual conditions. It also co-ordinates or integrates care provided elsewhere by others [21]. PHC is a conceptual model which refers to disease prevention, health promotion, population health, and community development within a holistic framework, with the aim of providing essential community-focused health care [22]. This is the level of Health Care we are studying in this article.

In Portugal, PHC has a history of about 40 years. Since there has been a constant need of implement reforms, it becomes necessary to understand how PHC evolved during the last 40 years. Therefore, it is important to distinguish two different periods: the first, between 1971 and 2004, when the first, second and third generation of health centers were created and the second one, from to 2005 up to now.

#### ***From 1971 to 2004***

The first legislation concerning health centers is from 1971, seven years before the Alma-Alta declaration. The “First Generation Health Centers” were created under the terms of the Decree-law 412/71. These Health Centers were responsible for ensuring public health: vaccination, women and childrend’s health, pregnancy care and health authority. At this time, the treatment of acute disease was not part of PHC activities. Such health care services were provided by the sickness insurance institutions [23].

Although seemingly paradoxical, regarding the health needs and patients’ expectations these two styles were complementary. That is the reason why a new legislation had been approved in 1983 (Decree-Law 97/83) which amended the preceding decree in order to integrate the health centers and the sickness insurance institutions, thus creating “Second Generation Health Centers”. However, this process was not totally peaceful, mainly due to the significant differences in the physical and human heritage. Because of that, consultations, home visits and health surveillance did not show the expected improvement. Additionally, these structures had very limited management autonomy [24].

To counteract this situation, a structural reorganization of PHC was provided for in 1999 (Decree-Law 157/99), which established an ideal model of a health center, the “Third Generation Health Centers”. However, in practice the situation of dependence was basically the same. This Decree-Law was repealed by the Decree-Law 60/2003, which aimed to improve the primary health care system by creating a network of healthcare provision. This network was supposed to improve the citizens’ evaluation capability. It was also an objective of the network to contribute to the reversal of the conservative policies and aversion to change apparently responsible for the inefficiency of the traditional health system. Nevertheless, this decree has never been implemented.

#### ***Since 2005***

A new phase of the Portuguese health sector started with the enactment of the Decree-Law 88/2005 which temporarily reinstated the one from 1999 and referred to a technical group specifically created to prepare the new health care reform. Since then the Government has begun studying ways of improving efficiency, equity and quality of PHC. During this period, some innovative ideas have arisen, the creation of ACES is an example of great importance.

By definition, the ACES are amalgamations of resources and management structures and are composed of different functional units. Their mission is to ensure the provision of PHC in a particular geographic area, enhancing health gains accomplished by Family Health Units (USFs) and providing better management structures. Also, the development of epidemiological surveillance activities, the research on health and the control and evaluation of results are inherent to the ACES’s mission. According to “The Mission for Primary Health Care”, many factors are taken into account when defining the geographic area of influence of the ACES: the number of residents that should be between 50,000 and 200,000, the population structure, the aging index and the accessibility to the reference hospital.

Each ACES is led by an Executive Director (DE) and consists of a Clinical Council (CC), a Community Council, a Management Support Unit (UAG) and five functional units, as described in Table 1. Other functional units might be considered, if the Health Regional Administration (ARS) decides so.

**Table 1:** The 5 types of functional units that compose the ACES

**Functional unit**	**Description**
USF	Individual and Family Care Unit. USFs promote the training of multidisciplinary teams, comprised by doctors, nurses and administrative staff. USFs allow a closer relationship with users through constant and personalized contact. In addition, in USFs all the patients have assigned doctors.
Personalized Healthcare Unit(UCSP)	Individual and Family Care Unit. In terms of dimension, it is similar to an USF. However, USFs are regulated by specific legislation, whereas UCSP are bounded to rules approved by the Clinic Council.
Community Care Unit (UCC)	UCCs provide care to groups with special needs and community interventions. UCCs operate in the community and are able of mobilizing skills inherent to other functional units, to provide health care through specific interventions.
Public Health Unit (USP)	USPs are related to population and environmental and public health. USPs are responsible for the planning and divulgation in public health. They are also in charge of epidemiological surveillance and manages population-wide programs in the domains of prevention, health promotion and protection.
Shared Assistential Resources Unit (URAP)	URAPs provide and enhance specific support and advice to the functional units and health projects of each ACES. Their mission is to support the former functional units.

**Figure 1:**
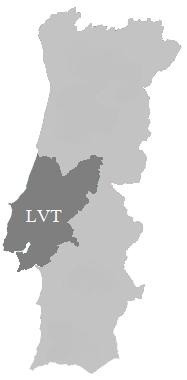
Location of Lisbon and Tagus Valley in the Portuguese territory.

Regarding the region of LVT (Figure 1), which corresponds to 13% of the Portuguese territory including the capital Lisbon, it has a population of 3,7 million (34% of the total population) and, according to the Centre for Regional Dynamics Observation (2009), it represents 44% of the national GDP. In this region, there are 22 ACES, organized according to the five existing NUTS III (subregions): 

• Grande Lisboa: Lisboa Norte (1), Lisboa Oriental (2), Lisboa Central (3), Oeiras (4), Odivelas (5), Loures (6), Amadora (7), Sintra - Mafra (8), Algueirão - Rio de Mouro (9), Cacém - Queluz (10), Cascais (11) and Vila Franca de Xira (12);

• Península de Setúbal: Almada (13), Seixal - Sesimbra (14), Arco Ribeirinho (15) and Setúbal - Palmela (16);

• Oeste: Oeste Norte (17) and Oeste Sul (18);

• Médio Tejo: Serra d’Aire (19) and Zêzere (20);

• Lezíria do Tejo: Ribatejo (21) and Lezíria II (22).

In the next chapter, the adopted methodology is presented: the non-parametric full frontier technique of DEA to study the efficiency of the ACES and the partial frontier technique of order-*m* so as to adjust the results obtained to the exogenous environment of each ACES.

## Methods

### Data collection

In this research, we analyzed the activity of the 22 ACES belonging to the LVT, mentioned at the end of Section ‘Background’, during the years 2009 and 2010. Most of the data used in this study was obtained from the ARS of LVT activity reports and the Project SimCidadão report. Both of the reports are freely available on-line. The data regarding staff was obtained from many contacts with the ARS of LVT. The use of this data required a previous authorization given by this organization. It is important to highlight that some inputs concerning costs were not available and that is the reason why most of the inputs are related to the staff. Table 2 shows the variables used as inputs and outputs, as well as the environmental variables. The input and output variables as well as the environmental variables were chosen taking into account the literature review and the available data. All the environmental variables were collected by analyzing the activity reports of the ACES.

**Table 2:** Inputs and Outputs used in the DEA models to measure efficiency and environmental variables

**Inputs**	**Outputs**	**Environmental variables**
*x*1: doctors’ working hours	*y*1: number of adult health consultations	*EV*1: Population
*x*2: nurses’ working hours	*y*2: number of speciality consultations	*EV*2: Population density
*x*3: administrative staff working hours	*y*3: number of urgency consultations (SAP,CATUS)	*EV*3: Percentage of patients aged 65 years old or older
	*y*4: number of home visits by doctors	*EV*4: Mortality Rate
*x*4: total costs	*y*5: total number of consultations	*EV*5: Percentage of patients withoutdesignated doctor
		*EV*6: Distance to the nearest hospital
	*y*6: number of group education sessions	*EV*7: Purchasing power
	*y*7: number of consultations by nurses	
	*y*8: number of injections, vaccinations, curatives and other treatments	
	*y*9: number of home visits by nurses	
	*y*10: total number of nursing services	
	*y*11: total number of public health activities	

### Data envelopment analysis

In order to study efficiency, we applied DEA, a non-parametric full frontier method based on linear programming technique with data from two consecutive years. This technique is used with the aim of evaluating the efficiency of the Decision Making Units (DMUs), by analysing the optimal combinations between inputs and outputs, i.e. between consumed resources and the resulting services or goods [25]. This methodology optimizes each individual observation and builds a production frontier, constituted by the efficient DMUs. Several models might be applied and choices regarding the type of return to scale and the orientation must be made in agreement with the production process that is being analyzed [26]. Concerning the orientation, we decided to use the following three orientations to compare the results and because in healthcare we can consider both points of view: 

• **Input orientation**: this orientation is used when it is assumed that we have more control of the inputs, it is also intended to emphasize the reduction of excessive inputs and we think it is possible to reach the same outputs with fewer inputs [27].

• **Output orientation**: the output-oriented model is adopted if one considers to have more control on the outputs, for instance, by controlling the reputation or the quality of service and when it is considered to be possible to increase the outputs, without any proportional change in the inputs [27].

• **Non-oriented model**: this model considers to be possible to reduce the inputs and simultaneously increase the outputs.

In this study, we used three possible orientations: input-orientation, output-orientation and also a non-oriented model, the additive one, which is based on slacks, excessive inputs or missing outputs that exists even after the proportional change in the input or the outputs [27]. These orientations were used in connection with both constant returns to scale (CRS) and variable returns to scale (VRS) technologies.

In the interest of understanding why both CRS and VRS technologies were used, it is important to clarify both concepts. CRS technology assumes that scale of economies do not change as the size of the service increases while VRS assumes that scale of economies do not change with the size of the service. Thus, CRS technology is not present if a proportional increase in one input can cause greater than a proportionate increase in output. In healthcare, not all of the DMUs are producing at the same scale, which might be due to many factors, such as financial constraints, technical constrains, or poor organization.

The method was chosen depending on the perspective of the analyst. If we believe that the DMU is not operating at the optimal scale, the VRS technology is recommended. However, by calculating both efficiencies, we can determine a third efficiency measure: the scale efficiency, which is defined as the ratio between the efficiency computed with between VRS and CRS:

(1) $$\theta_{\text{scale}}=\theta_{\text{VRS}}/\theta_{\text{CRS}}$$

The efficiency scores (*θ*) for the DMUs (*j*=1,…,*n*) are computed for the selected outputs (*y*_*rj*_) and inputs (*x*_*ij*_). We used the dual model in order to observe benchmarks and their weights (*λ*). The (*λ*) values allow us to understand the return to scale for each DMU. If *Σλ*=1, it means that the DMU is working with CRS. If *Σλ*<1, the DMU is working with increasing returns to scale. Finally, if *Σλ*>1, the decreasing return to scale is the technology. In the objective function (*ε*) is called the non-Archimedean, which is defined as infinitely small. *s*^-^ and *s*^+^ represent input and output slacks, respectively (Table 3).

**Table 3:** **Mathematical formulation of the different models and orientations [**[27]**]**

**Orientation**	**CRS**	**VRS**
Inputs	$\text{Minimize}\;\theta -\varepsilon (\overset{m}{\underset{i=1}{\sum }}s_{i}^{-}+\overset{s}{\underset{r=1}{\sum }}s_{r}^{+})$ subject to 1a. $\overset{n}{\underset{j=1}{\sum }}\lambda_{j}x_{\text{ij}}+s_{i}^{-}=\theta^{*}x_{i0}$ with *i*=1,…,*m* 2a. $\overset{n}{\underset{j=1}{\sum }}\lambda_{j}y_{\text{rj}}-s_{r}^{+}=y_{r0}$ with *r*=1,…,*s* 3. *λ*_*j*_≥0 with *j*=1,…,*n*	$\text{Minimize}\;\theta -\varepsilon (\overset{m}{\underset{i=1}{\sum }}s_{i}^{-}+\overset{s}{\underset{r=1}{\sum }}s_{r}^{+})$ subject to 1a, 2a, 3 and 4. $\overset{n}{\underset{j=1}{\sum }}\lambda_{j}=1$
Outputs	$\text{Maximize}\;\phi -\varepsilon (\overset{m}{\underset{i=1}{\sum }}s_{i}^{-}+\overset{s}{\underset{r=1}{\sum }}s_{r}^{+})$ subject to 1b. $\overset{n}{\underset{j=1}{\sum }}\lambda_{j}x_{\text{ij}}+s_{i}^{-}=x_{i0}$ with *i*=1,…,*m* 2b. $\overset{n}{\underset{j=1}{\sum }}\lambda_{j}y_{\text{rj}}-s_{r}^{+}=\phi y_{r0}$ with *r*=1,…,*s* 3. *λ*_*j*_≥0 with *j*=1,…,*n*	$\text{Maximize}\;\phi -\varepsilon (\overset{m}{\underset{i=1}{\sum }}s_{i}^{-}+\overset{s}{\underset{r=1}{\sum }}s_{r}^{+})$ subject to 1b, 2b, 3 and 4. $\overset{n}{\underset{j=1}{\sum }}\lambda_{j}=1$
Non-oriented	$\text{Maximize}\;\overset{m}{\underset{i=1}{\sum }}s_{i}^{-}+\overset{s}{\underset{r=1}{\sum }}s_{r}^{+}$ subject to 1c. $\overset{n}{\underset{j=1}{\sum }}\lambda_{j}x_{\text{ij}}+s_{i}^{-}=x_{i0}\;i=1,\ldots ,m$ 2c. $\overset{n}{\underset{j=1}{\sum }}\lambda_{j}y_{\text{rj}}-s_{r}^{+}=y_{r0}\;r=1,\ldots ,s$ 3. *λ*_*j*_≥0 *j*=1,…,*n*	$\overset{m}{\underset{i=1}{\sum }}s_{i}^{-}+\overset{s}{\underset{r=1}{\sum }}s_{r}^{+}$ subject to 1c, 2c, 3 and 4. $\overset{n}{\underset{j=1}{\sum }}\lambda_{j}=1$

In contrast with the parametric techniques, such as the Stochastic Frontier Analysis, the non-parametric technique of DEA seems to be a better option since it does not require the specification of the functional form that links the inputs to the outputs.

#### ***Adopted models***

In the light of data constraints, 4 models were used: medicine, nursing, global and global with total costs. They are presented next: **Model I (Medicine):** it includes all the ACES’ medical services. The input used was *x*1 and the outputs were *y*1, *y*2, *y*3 and *y*4; **Model II (Nursing):** it comprises all the ACES’ nursing services. The input adopted was *x*2 and the outputs were *y*6, *y*7, *y*8 and *y*9; **Model III (Global):** this model aims to include all the ACES’ services. The input used was *x*1, *x*2 and *x*3 and the outputs were *y*5, *y*10 and *y*11; **Model IV (Global with total costs):** model similar to Model III. However, the input adopted was *y*4 (total costs), instead of staff number resources. The outputs used were *y*5, *y*10 and *y*11.

These models were used in order to include the two main services of the ACES (medicine and nursing service) and to calculate the global efficiency. The global efficiency is calculated by using two different models because the data regarding the costs was only available for the second year of study.

### The order-m methodology

The environmental variables are exogenous factors that cannot be categorized as inputs or outputs but might affect efficiency [28]. As a result, they are not controlled by the DMU managers. It is extremely important to take these variables into account. If not, biased conclusions might be drawn. Although there is no agreement on the best technique to be used, in this study we used a recent methodology: the order-*m*[28], because, being a partial frontier method, order-m has proven to be less sensitive than DEA to extreme values and able to overcome the deterministic nature of traditional non-parametric techniques [28]. According to the order-*m* methodology, the production process can be described by the joint probability of measure of ≪(*X*,*Y*), on space ${ℜ}_{+}^{p}\times {ℜ}_{+}^{q}$ according to 2.

(2) $$H_{\text{XY}}(x,y)=\text{Prob}(X\leq x,Y\geq y)$$

where *X* are the inputs and *Y* are the outputs.

Afterwards, in an input orientation context, the likelihood function can be decomposed into two according to Bayes rule (3), and then, efficiency can be computed (4, 5, 6).

(3) $$\begin{array}{c} H_{\text{XY}}(x,y)=\mathrm{Prob}(X\leq x|Y\geq y)\mathrm{Prob}(Y\geq y)\\ \;=F_{X|Y}(x|y)S_{Y}(y) \end{array}$$

(4) $$\begin{array}{c} \theta (x,y)=\inf\{\theta |F_{X|Y}(\mathrm{\theta x}|y)>0\}\\ \;=\inf\{\theta |H_{\text{XY}}(\mathrm{\theta x},y)>0\} \end{array}$$

(5) $$\hat{\theta }(x,y)=\inf\{\theta |{\hat{F}}_{X|Y}(\mathrm{\theta x}|y)>0\}$$

(6) $${\hat{\theta }}_{m,n}(x,y)=\overset{\infty }{\underset{0}{\int }}{\left(1-{\hat{F}}_{X|Y,n}(\text{ux}|y)\right)}^{m}du$$

with ${\hat{F}}_{X|Y,n}(\text{ux}|y)=\frac{\overset{n}{\underset{i=1}{\sum }}I(X_{i}\leq \text{ux},Y_{i}\geq y)}{\overset{n}{\underset{i=1}{\sum }}I(Y_{i}\geq y)}$ and *I*(*k*) being the indicator function that take the value *I*(*k*)=1 when *k* is true or *I*(*k*)=0 otherwise.

According to Daraio and Simar [28], the inclusion of exogenous variables can easily be done by limiting the production process to a given value of the exogenous variable (7, 8).

(7) $$H_{\text{XY}}(x,y)=F_{X|Y,Z}(x|y,z)S_{Y|Z}(y|z)$$

(8) $${\hat{\theta }}_{m}(x,y|z)=\overset{\infty }{\underset{0}{\int }}{\left(1-{\hat{F}}_{X|Y,Z,n}(\text{ux}|y,z)\right)}^{m}du$$

where ${\hat{F}}_{X|Y,Z,n}(\text{ux}|y,z)=\frac{\overset{n}{\underset{i=1}{\sum }}I(X_{i}\leq \text{ux},Y_{i}\geq y)K\left(\frac{\left(Z-Z_{i}\right)}{h}\right)}{\overset{n}{\underset{i=1}{\sum }}I(Y_{i}\geq y)K\left(\frac{Z-Z_{i}}{h}\right)}$, *h* is the bandwidth and *K*(∙) is the kernel function.

With the aim of analyzing the influence of the exogenous variable on the production process, a non-parametric smoothed regression of the ratios between the order-m conditional efficiencies and the unconditional efficiencies is applied. If the regression has a positive slope, the exogenous variable has a negative effect on the production process because the environmental variable acts like an “undesirable” output to be produced, requiring the usage of more inputs [28]. Otherwise, it will have the opposite effect.

### The Kruskal-Wallis test

An important study regarding the PHC is the analysis of the horizontal equity, which concerns fairness or justice in the treatment and measures whether patients from different groups have similar access to the services they equally need [13].

In order to analyze the horizontal equity of access regarding the NUTS III we studied the relationship between the NUTS III and (1) the efficiency and (2) the percentage of patients without a designated doctor. To test the hypothesis of relevant differences in equity, we used the non-parametric *Kruskal-Wallis* test, with multiple comparisons.

The *Kruskal-Wallis* test is a non-parametric method used to determine whether three or more independent groups are the same or different on some variable of interest [29]. It calculates the probability of being wrong when concluding that there is no difference between three or more treatment groups [30].

The test will assume that the samples are identical and check if the differences found between the groups are genuine and if there are evidences to reject the null hypothesis.

The ANOVA test is more powerful if we are studying a normal distribution. However, since we are using a non-parametric method, the Kruskal-Wallis is more appropriate because it does not assume that kind of distribution.

## Results

### Sample characteristics

The summary statistics for the variables used in the research are described in Table 4.

**Table 4:** Summary statistics of inputs and outputs (year 2010)

**Variables**	**Average**	**Evolution**	**Median**	**Std. deviation**	**Maximum**	**Minimum**
		**(2009 to 2010)**				
*x*1	3583	-3.9%	3596	1191	6009	1750
*x*2	3537	-1.6%	3693	706	4655	2170
*x*3	6116	-5.8%	5828	1709	9905	2660
*x*4	34147052	ND	34817334	7503483	44872810	17898835
*y*1	351901	1.8%	355779	79532	517515	189954
*y*2	75365	-0.1%	73766	26072	158988	39745
*y*3	47693	-24.24%	47052	32488	105067	2503
*y*4	1879	3.4%	1788	932	4976	576
*y*5	476837	-1.9%	451655	109922	685802	276495
*y*6	918	-5.3%	726	868	1804	1
*y*7	212289	9.3%	181902	80977	485108	107244
*y*8	173392	0.7%	147439	73976	317073	78673
*y*9	50368	4.0%	42213	21870	103511	28695
*y*10	436967	5.1%	396031	148284	842544	237951
*y*11	13907	-0.9%	10060	10309	39012	3876

### Efficiency and environmental variables

Both CRS and VRS technologies concerning input orientation, output orientation and the non-oriented model were used in order to compare the results from each approach. Tables 5, 6 and 7 summarize the efficiency results for the three kinds of orientation.

**Table 5:** Efficiency results for 2009 and 2010 (input orientation)

	**Model I**		**Model II**		**Model III**		**Model IV**
**Year**	**2009**	**2010**	**2009**	**2010**	**2009**	**2010**	**2010**
*θ*_*CRS*_ average	0.793	0.816	0.764	0.769	0.913	0.935	0.918
min *θ*_*CRS*_	0.581	0.569	0.469	0.465	0.761	0.817	0.745
DMU with min *θ*_*CRS*_	2	2	21	11	11	11	2
Number of efficient DMUs (CRS)	4	6	5	6	8	8	6
DMUs with *θ*_*CRS*_>95*%*	4	6	6	7	9	11	12
DMUs with *θ*_*CRS*_<60*%*	2	1	5	3	0	0	0
Most common benchmarks (CRS)	22	22	17	17	17	17	8
*θ*_*VRS*_ average	0.852	0.857	0.839	0.849	0.944	0.961	0.928
min *θ*_*VRS*_	0.585	0.595	0.581	0.520	0.775	0.835	0.754
DMU with min *θ*_*VRS*_	11	2	21	15	11	15	2
Number of efficient DMUs (VRS)	7	8	8	7	11	13	9
DMUs with *θ*_*VRS*_>95*%*	9	8	8	9	13	15	12
DMUs with *θ*_*VRS*_<60*%*	1	1	1	1	0	0	0
Most common benchmarks (VRS)	15	13 e 22	5	17	17	13 e 17	14
*θ*_*Scale*_ average	0.935	0.954	0.905	0.903	0.968	0.972	0.990
min *θ*_*Scale*_	0.722	0.783	0.690	0.622	0.837	0.848	0.960
DMU with min *θ*_*Scale*_	15	17	9	9	12	5	9
Number of scale efficient DMUs	4	6	5	6	8	8	7
DMUs with *θ*_*Scale*_>95*%*	11	17	8	10	17	17	22
DMUs with *θ*_*Scale*_<60*%*	0	0	0	0	0	0	0

**Table 6:** Efficiency results for 2009 and 2010 (output orientation)

	**Model I**		**Model II**		**Model III**		**Model IV**
**Year**	**2009**	**2010**	**2009**	**2010**	**2009**	**2010**	**2010**
*θ*_*CRS*_ average	1,3010	1,264	1,383	1,380	1,104	1,076	1,099
max *θ*_*CRS*_	1,722	1,758	2,132	2,149	1,314	1,224	1,343
DMU with max *θ*_*CRS*_	2	2	21	11	11	11	2
Number of efficient DMUs (CRS)	4	6	5	6	8	8	6
DMUs with *θ*_*CRS*_<1,05	4	6	6	7	9	11	12
DMUs with *θ*_*CRS*_>1,6	3	3	6	5	0	0	0
Most common *Benchmark*	22	22	17	17	17	17	8
*θ*_*VRS*_ average	1,154	1,134	1,307	1,292	1,049	1,037	1,084
max *θ*_*VRS*_	1,585	1,590	2,125	2,017	1,306	1,188	1,3367
DMU with max *θ*_*VRS*_	5	5	21	11	11	11	2
Number of efficient DMUs (VRS)	7	8	8	7	11	13	9
DMUs with *θ*_*VRS*_<1,05	10	10	8	9	14	15	12
DMUs with *θ*_*VRS*_>1,6	0	0	4	4	0	0	0
Most common *Benchmark*	17	13	17	17	17	17	8 e 14
*θ*_*Scale*_ average	1,132	1,115	1,062	1,070	1,053	1,037	1,014
max *θ*_*Scale*_	1,432	1,412	1,449	1,607	1,189	1,179	1,094
DMU with max *θ*_*Scale*_	15	1	9	9	1	5	15
Number of scale efficient DMUs	4	6	5	6	8	8	6
DMUs with *θ*_*Scale*_<1,05	9	10	13	12	14	16	21
DMUs with *θ*_*Scale*_>1,6	0	0	0	1	0	0	0

**Table 7:** Efficiency results for 2009 and 2010 (non-oriented model)

	**Model I**		**Model II**		**Model III**		**Model IV**
**Year**	**2009**	**2010**	**2009**	**2010**	**2009**	**2010**	**2010**
Number of efficient DMUs (CRS)	4	6	5	6	8	8	6
Number of non efficient DMUs (CRS)	18	16	17	16	14	14	16
Most common *Benchmark*	22	22	17	17	17	17	8
Number of efficient DMUs (VRS)	7	8	8	7	11	13	9
Number of non efficient DMUs (VRS)	15	14	14	15	11	9	13
Most common *Benchmark*	13	13	17	17	17	17	14

Tables 8, 9 and 10 shows the *λ* results analysis, *i.e.*, the benchmarks and also the returns to scale, as explained in Subsection ‘Data envelopment analysis’.

**Table 8:** General statistics concerning returns to scale (input orientation)

	**Model I**		**Model II**		**Model III**		**Model IV**
**Year**	**2009**	**2010**	**2009**	**2010**	**2009**	**2010**	**2010**
*Σλ*_*CRS*_ average	1.393	1.310	0.753	0.829	0.857	0.945	0.942
max *Σλ*_*CRS*_	3.506	2.941	1.137	1.284	1.123	1.488	1.316
DMU with max *Σλ*_*CRS*_	3	3	1	16	21	11	16
# of DMUs with *Σλ*_*CRS*_>1	14	12	2	4	3	6	6
min *Σλ*_*CRS*_	0.862	0.941	0.362	0.486	0.473	0.518	0.523
DMU with min *Σλ*_*CRS*_	12	5	9	9	9	5	9
# of DMUs with *Σλ*_*CRS*_<1	4	4	15	12	11	8	10
# of DMUs with *Σλ*_*CRS*_=1	4	6	5	6	8	8	6

**Table 9:** General statistics concerning returns to scale (output orientation)

	**Model I**		**Model II**		**Model III**		**Model IV**
**Year**	**2009**	**2010**	**2009**	**2010**	**2009**	**2010**	**2010**
*Σλ*_*CRS*_ average	1,798	1,672	2,038	1,520	0,938	1,013	1,028
max *Σλ*_*CRS*_	3,721	3,433	7,018	3,207	1,288	1,822	1,465
DMU with max *Σλ*_*CRS*_	3	3	14	14	15	11	1
# of DMUs with *Σλ*_*CRS*_>1	18	16	15	15	6	7	10
min *Σλ*_*CRS*_	1	1	0,884	0,781	0,483	0,581	0,544
DMU with min *Σλ*_*CRS*_	-	-	9	9	9	12	9
# of DMUs with *Σλ*_*CRS*_<1	0	0	2	1	8	7	6
# of DMUs with *Σλ*_*CRS*_=1	4	6	5	6	8	8	6

**Table 10:** General statistics concerning returns to scale (non-oriented model)

	**Model I**		**Model II**		**Model III**		**Model IV**
**Year**	**2009**	**2010**	**2009**	**2010**	**2009**	**2010**	**2010**
*Σλ*_*CRS*_ average	1,832	1,761	0,9812	0,938	0,939	0,932	0,9418
max *Σλ*_*CRS*_	3,581	3,293	1,378	1,406	1,306	1,207	1,316
DMU with max *Σλ*_*CRS*_	3	3	18	3	15	15	16
# of DMUs with *Σλ*_*CRS*_>1	18	16	8	5	6	5	6
min *Σλ*_*CRS*_	-	-	0,537	0,5345	0,483	0,5628	0,5225
DMU with min *Σλ*_*CRS*_	-	-	9	9	9		9
# of DMUs with *Σλ*_*CRS*_<1	0	0	9	11	8	9	10
# of DMUs with *Σλ*_*CRS*_=1	4	6	5	6	8	8	6

**Figure 2:**
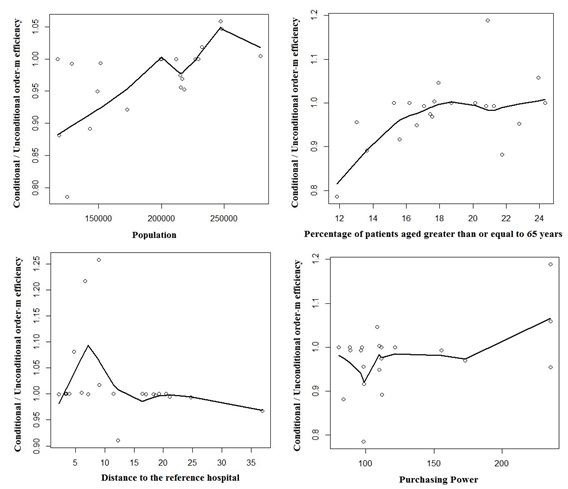
Influence of the exogenous variables in 2009.

**Figure 3:**
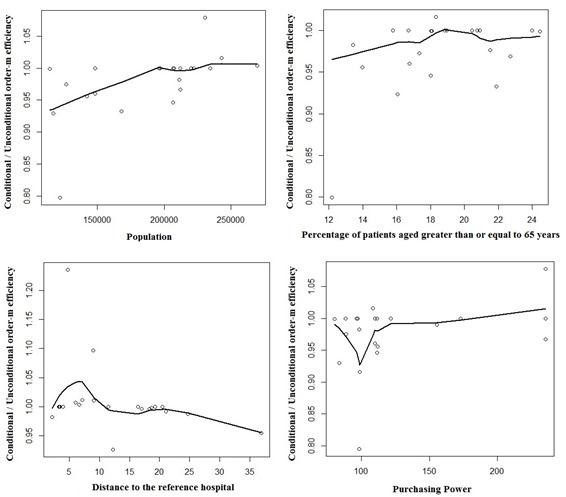
Influence of the exogenous variables in 2010.

Regarding the environmental variable, the graphic results of the order-m regression are illustrated in Figures 2 and 3.

### Equity of access

**Figure 4:**
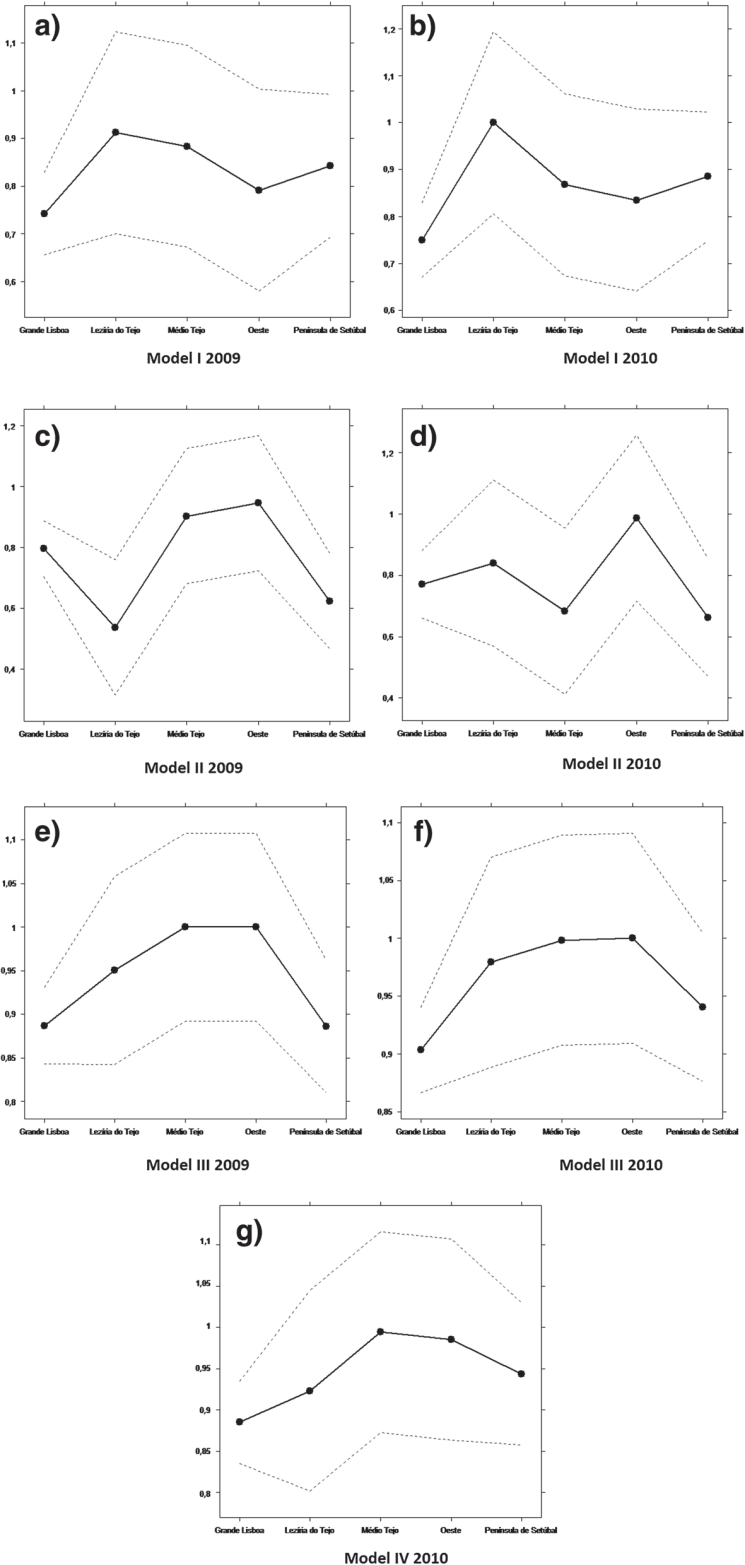
**Results of the first Kruskal-Wallis test for input oriented CRS (*α*** **=0** ***.*** **05 and confidence interval = 95 %), regarding Model I in 2009 (a) and 2010 (b), Model II in 2009 (c) and 2010 (d), Model III in 2009 (e) and 2010 (f) and regarding Model IV in 2010 (g).**

**Figure 5:**
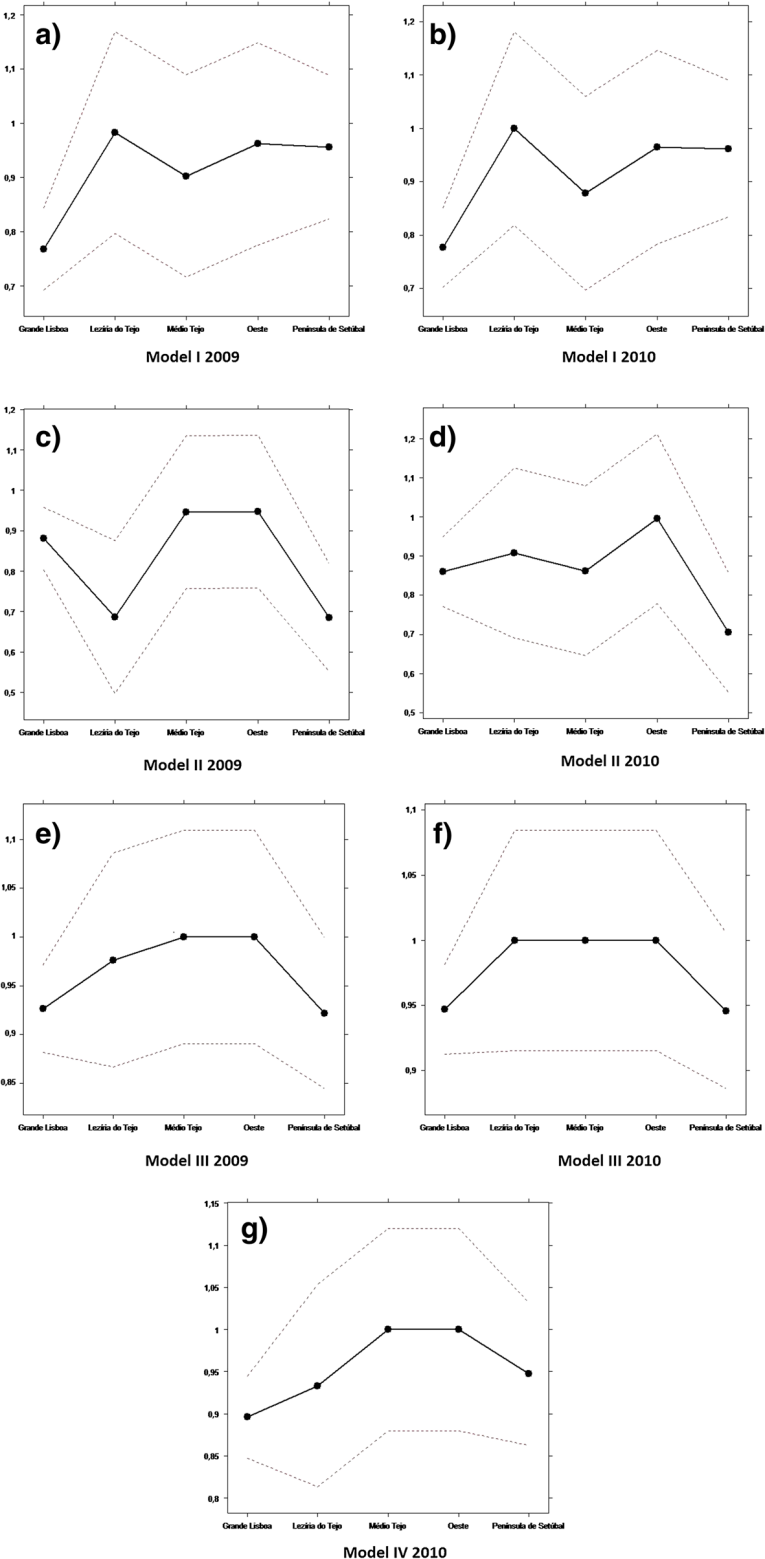
**Results of the first Kruskal-Wallis test for input oriented VRS (*α*** **=0** ***.*** **05 and confidence interval = 95%), regarding Model I in 2009 (a) and 2010 (b), Model II in 2009 (c) and 2010 (d), Model III in 2009 (e) and 2010 (f) and regarding Model IV in 2010 (g).**

Figures 4 and 5 show the results of the first Kruskall-Wallis test: the relationship between the NUTS III and the efficiency.

**Figure 6:**
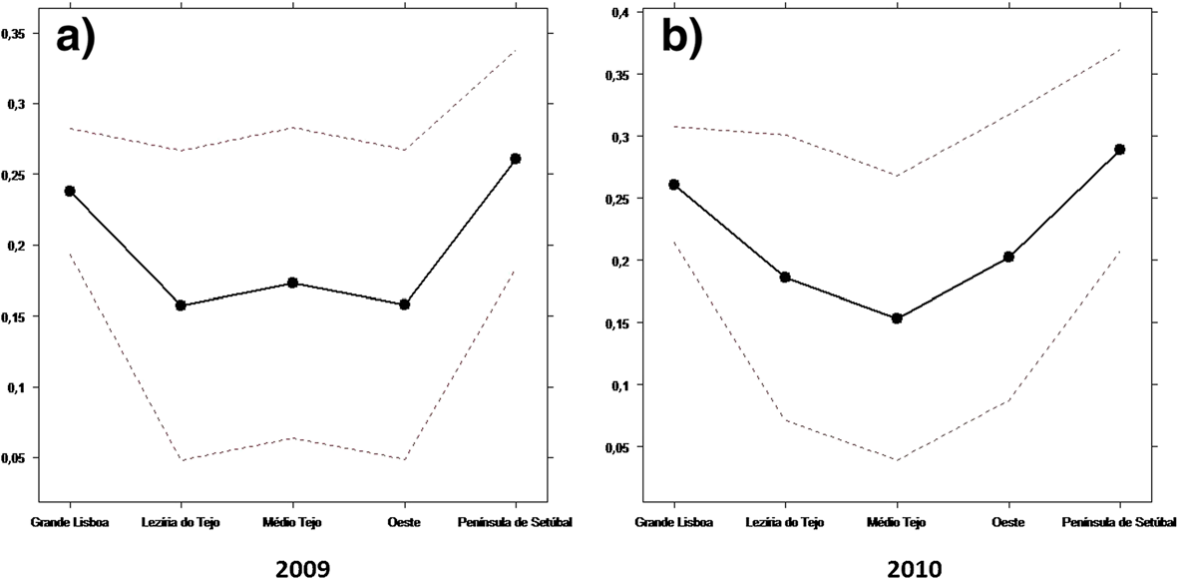
Results of the second Kruskal-Wallis test for the years 2009 (a) and 2010 (b).

Figure 6 shows the results of the second test: the relationship between the NUTS III and the percentage of patients without a designated doctor.

### Quality

**Figure 7:**
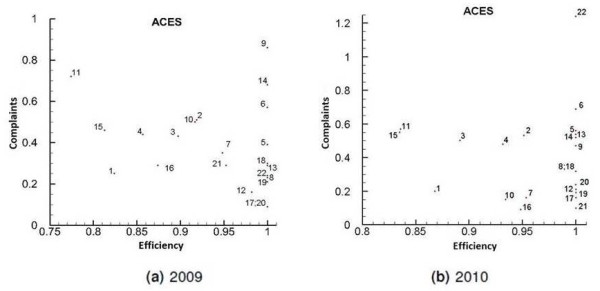
Trade-offs between efficiency and complaints for the years 2009 (a) and 2010 (b).

In addition to the efficiency and the equity of access, it is also of utmost importance to evaluate the quality of the services provided. According to Fornell [31], the patients’ level of satisfaction on the PHC is very important to track the quality of service. Therefore, this analysis uses the ratio between the complaints and the total of ACES’ activities. These data are reported in the Project SimCidadão report and can be considered a good indicator of the patient’s level of satisfaction. Figure 7 shows the trade-offs between the VRS efficiency and the ratio addressed above, for the years 2009 and 2010.

## Discussion

### Efficiency and the influence of the environment on the efficiency

By analyzing the general efficiency results referred to in Subsection ‘Quality’ (Tables 5, 6 and 7 some comments can be made. In general, we can see that the results were very similar for all types of used orientation.

Regarding the results themselves, the ACES 17 (Oeste Norte) is one of the most efficient. As can be seen in Table 5, this ACES is referred to 7 times as the most common benchmark, i.e. the best practice for the other ACES. In fact, Model I (CRS) is the only model where this ACES does not provide a unitary efficiency. ACES 2 and 11 are the least efficient. In fact, these ACES have poor results regarding contractualization. The model with the lowest efficiency is Model II (Nursing), where the number of scores below 60% that can be found is maximum. A general improvement of efficiency between 2009 and 2010 can be observed. For instance, in 2009, VRS showed a result of 0.944 for Model III, while in 2010, the same technology showed a score of 0.96. One of the possible reasons is the start-up of 10 new USFs. Also, 2009 was the foundation year of the ACES and it is likely that they were not as organized as in 2010.

Regarding the returns to scale, some important conclusions can also be drawn. By observing Table 8 (and also Tables 9 and 10), it can be seen that as far as Model I is concerned, more than half of the ACES are working with decreasing returns to scale, i.e. they are operating above the optimal scale. ACES 1 and 3 always present decreasing returns to scale for Model I. Actually, both ACES are serving a population over the theoretical limit of 200 000. Probably the effect of diseconomies of scale would decrease if the 3 ACES of the Lisbon area (Lisboa Norte, Lisboa Oriental and Lisboa Central) were split into 4. In contrast, in Model II (nursing), more than 50% of the ACES have increasing returns to scale. This means that concerning the variables studied, in these ACES nursing is operating below the optimal scale.

As stated before, adjusting for the environment in efficiency studies is very important. In this paper, the exogenous variables considered are the ones described in Table 3, respectively population density, percentage of patients aged 65 years old or older, mortality rate, percentage of patients without a designated doctor, distance to the nearest hospital and purchasing power.

Since the sample includes only ACES from LVT, without considering other important variables, such as laws and policy administration, they can be considered relatively homogeneous. It is important to highlight the meaning of variable 6, the distance to the reference hospital. This distance refers to the average of the distances between the head office of each health center and the reference hospital.

The discussion of the results obtained by using the order-m methodology is summarized below.

Concerning the population, efficiency decreases with the increase of the population. This result suggests that the population acts as an output, requiring more inputs in the production process. This is in agreement with the suggestion made before, about splitting the 3 ACES of Lisbon into 4.

As to the population density, the results show that there is no influence on efficiency.

About the percentage of patients aged 65 years old or older, there is a negative influence of this variable on efficiency. In fact, aging leads to a significant rise in the need for community and social care. Consequently, efficiency decreases due to the increase of costs associated with aging.

Mortality shows no influence on efficiency. Avoidable mortality could have been used instead. However, there were no data available on this variable.

As far as the percentage of patients without a designated doctor is concerned there is no influence of this variable on efficiency, maybe because of the reorganization of some of the functional units of the ACES, namely USFs and UCSPs.

The variable distance to the reference hospital has a positive influence on efficiency. This is not surprising, as when a hospital is located farther away from a population, more patients will go to a PHC.

Regarding purchasing power, this variable has a negative influence on the efficiency. Actually, patients with higher purchasing power tend to have health insurance and use the PHC less. Furthermore, patients with less purchasing power cannot afford private health care and need to resort to PHC.

### Equity of access

By observing Figures 4, 5 and 6, we see that both tests suggest that there is no evidence of relevant differences, and consequently there is horizontal equity regarding the NUTS III. In fact, despite some demographic inequalities, all the municipalities have healthcare facilities. The USFs can contribute to improve equity because one of their main goals is to have close contact with the population and to avoid having patients without a designated doctor [32].

These results go in line with the study from a public authority (Alto Comissariado da Saúde [33]). This study states that an access improvement, a higher number of consultations and a bigger rational of healthcare have been observed since 2009.

### Quality

Figure 7 shows a large number of complaints in Grande Lisboa and Península de Setúbal. However, this behavior might be a consequence of patients’ higher expectations. Because they have many additional options, they might expect a different kind of service. Also, in Figure 7 some particular results should be noticed. There is a higher incidence of complaints for ACES Algueirão - Rio de Mouro (9) in 2009 and for ACES Lezíria II (22) in 2010. Regarding the ACES Lezíria II (22), an increase of about 200% was recorded, compared with 2009. This increase may be due to the lack of doctors and the excess work load of the doctors working in this ACES. The reason why in the ACES Algueirão - Rio de Mouro (9) there was a higher incidence of complaints is probably the same. In fact, this is the ACES with higher average number of patients per doctor. The ACES Cascais (11) and Arco Ribeirinho (15) can be found in the region of high complaints and low efficiency, for both years. The high level of complaints of ACES 15 may be related to the average waiting time for a consultation in a UCSP, which is typically greater than the average waiting time in a USF.

Finally, the ACES Oeste Norte (17) presents one of the highest patient levels of satisfaction. This ACES is the one that can be used as a benchmark for most of other ACES, as previously mentioned. This idea is also corroborated by the fact that the average number of patients per doctor is low.

It is important to stress that the rate of complaints is only an indicative measure of the patients’ satisfaction level. The rate of complaints might be used as a guideline to satisfaction and quality, but satisfaction surveys would have been better in our view. Although ARS has been trying to carry out these surveys, they are currently suspended as a result of the current economic conditions.

## Conclusions

Taking into account the pressure to decrease costs with healthcare, the efficiency analysis of PHC is of paramount importance. However, besides the problems with getting data, the evaluation of PHC is still a difficult and controversial task. Nonetheless, during this analysis, some important conclusions were drawn.

In terms of efficiency, on the whole, there was a general improvement in efficiency between 2009 and 2010. It was found that nursing was the service with lowest scores. In particular, the ACES Oeste Norte (17) was scored as one of the most efficient and identified as a unit to be used as benchmark. On the other hand, ACES Lisboa Oriental (2) and Cascais (11) were scored as the least efficient. With regard to returns to scale, it would be a good policy to split the 3 ACES from Lisbon (Lisboa Norte, Lisboa Oriental and Lisboa Central) into 4. Regarding equity of access, the objectives of ensuring equal opportunity to all the patients and the allocation of resources and services in a fair, consistent and inclusive manner appears to be working well, at least according to the two tests performed.

Concerning quality versus efficiency, the ACES which can more successfully combine both these aspects are the ACES Zêzere (20), Ribatejo (21) and also the ACES Oeste Norte (17), previously referred to as a benchmark. Conversely, the most problematic cases are the ACES Cascais (11) and Arco Ribeirinho (15), both located in the region of high complaints and low efficiency. Other examples of problematic cases are the ACES Algueirão - Rio de Mouro (9) and Lezíria II (22). These ACES are efficient only because they have a low level of human resources and because of that they receive massive complaints. As previously mentioned, satisfaction surveys regarding PHC are, at least for the time being, suspended. We intend to include them in this study as soon as they become available.

Finally, regarding the exogenous variables, the distance to the nearest hospital has a positive influence on efficiency. In contrast the order-m test showed that the purchasing power, the percentage of patients aged 65 years old or older and the population affect efficiency negatively.

In order to strengthen this study, it should be repeated with more information and more robust data in the future. For instance, it would be important to include more data regarding costs and health results, such as prevention and control of diabetes. The inclusion in the study of economies of scope would also be a good complement to this work. Also, it would be interesting to extend this study to all the ACES and even compare the efficiency of PHC before and after the reform of the PHC structure.

Nevertheless, despite some difficulties and some work that can still be done, this research is important to understand how the ACES have been performing in their first years of activity. This study shows that by establishing a learning network there is room for improving the efficiency of ACES with a better usage of the available resources whilst simultaneously keeping quality and equity.

Finally, we should note that an important aspect in this kind of studies is the fact that they allow the assessment of the current structure of the PHC in the country while taking into account not only the size but also other factors which are important for their performance, such as the proximity of hospitals.

## Competing interests

There are no competing interests, financial or non-financial.

## Authors’ contributions

CF was responsible for acquisition of data, carried out the study, interpreted the results and drafted the manuscript. RCM participated in the design and coordination of the study, provided technical help regarding the methodology and helped to review all the manuscript. PN participated in the analysis and interpretation of results and revised all the manuscript from a medical point of view. All authors read and approved the final manuscript.

## Pre-publication history

The pre-publication history for this paper can be accessed here:

http://www.biomedcentral.com/1472-6963/13/529/prepub
